# The School Doctor and the Mentally Defective Child

**Published:** 1916-05-27

**Authors:** 


### THE SCHOOL DOCTOR.

### The School Doctor and the Mentally Defective Child.

The passing of the Mental Deficiency Act of 1913, and the compulsory adoption of the Defective and Epileptic Children Act in all areas, has had the effect of making school hygiene a very important part of psychological medicine. The duties of the school medical officer now include the certification of all aments under sixteen, supervision of the education of such as are educable, and in some districts he is also asked to deal with adult aments in prisons, workhouses, etc. The proper performance of these tasks obviously demands some acquaintance with child psychology and educational problems, as well as psychiatry; and the work is further complicated by a series of overlapping legal definitions on whose meaning no two experts agree, and a hopelessly confused nomenclature. The most important section of this medico-psychological work deals with feeble-minded of school age, technically known as "mentally defective children." The statutory duty of every local education authority is to ascertain what children within its area having attained the age of seven are incapable of deriving benefit from instruction in an ordinary elementary school, but are capable of deriving benefit from a special school or class; and, further, to provide suitable education for such children under the age of sixteen (Defective and Epileptic Children Act, 1908).

It is obvious that as the legal definition here gives a scholastic test of defect, the duty of selecting suitable children for certification must fall in the first instance on the teachers. Much of the success of the whole scheme depends on this primary selection, and it is of the utmost importance that teachers, higher education officials, and the certifying school doctor should work together in close agreement as to the class of case suitable for special education. Left to themselves, teachers tend to withhold the most suitable children on the ground that special schools are for "sillies," or else they attempt to use them as a dumping ground for all kinds of juvenile undesirables, quite irrespective of defects in intelligence.

A good working rule suggested by M. Binet is to refer all children two or more years retarded before the age of nine, or three years retarded after that age, for special examination by the doctor, who fixes his standard on the basis that the highest-grade children who leave between fourteen and sixteen should be doing Standard II. work and should test at not more than the eleven-year grade in general intelligence. It is the opinion of Dr. Tredgold and most other experts that children above this level are capable of progress in the ordinary schools. The lower limit of suitability is less easy to define, and there is much difference in theory and practice, the Acts giving little guidance. The doctor has further to contend with pathetic parental blindness, with sentimental busybodies, and even with curious misunderstandings on the part of members of his own profession. Quite recently two children were sent from the out-patient department of a great hospital with peremptory written demands to the school authorities for immediate admission to special schools. One was a destructive idiot boy of seven who could only utter animal cries; the other an imbecile girl of eight who could not say her own name and greeted simple commands with a vacant grin. The admission of children of this type is an abuse of public money and a waste of teachers' talents, which revolts the common sense of the public and ends in the discredit of the whole question of the education of defectives. Children with intractably dirty habits or serious moral perversions are more fitted for residential institutions than for day schools, and no child should be retained unless he is making definite improvement greater than he would make if kept in his own home.

The certifying doctor will find his work greatly facilitated if some official of the local Education Department assists at his examination. In Liverpool the Superintendent of Special Schools is always present, elsewhere the Director of Education or a senior head-teacher gives his services. The object of the inquiry is not to perform an elaborate psychological investigation, but rather to confirm the teacher's report on the child's scholastic attainments, to eliminate causes of spurious defect such as malnutrition, bad attendance, defect of the special senses, and lastly to test the "general intelligence." Here the Binet-Simon scale, modified by Goddard, and supplemented by such further tests as the experience of the examiner suggests, will be found invaluable. The cardinal point to be elicited is a comparison with a normal child *of the same age*, and without a standardised scale for a basis the examiner is utterly at sea whatever his previous experience. A very cocksure inspector has been heard to exclaim, on seeing a twelve-year-old child doing seven-year-old tests rather badly, "If that child is defective, then I am!" It must be emphasised that a diagnosis of mental defect will, of course, depend on the whole mental and physical record of the case, and not on the response to any one query or series of queries. Hence the necessity for insisting that the final decision must be in the hands of the doctor, and not of a layman.

